# An improved VSG control strategy based on transient electromagnetic power compensation

**DOI:** 10.1038/s41598-023-42402-9

**Published:** 2023-09-12

**Authors:** Changwei Gao, Wei Wang, Chongyang Huang, Weiqiang Zheng

**Affiliations:** 1 College of Electrical and Automation Engineering, Liaoning Institute of Science and Technology, Benxi, CO 117004 China; 2 School of Electrical Engineering, Shenyang University of Technology, Shenyang, CO 110870 China; 3 Yingkou Power Supply Company, Yingkou, CO 115002 China

**Keywords:** Electrical and electronic engineering, Photovoltaics

## Abstract

Virtual synchronous generator (VSG) not only increases the inertia of grid-connected system, but also brings the problem of active power oscillation under grid disturbance. Therefore, VSG control strategy and system model order reduction method with transient electromagnetic power compensation are proposed. The closed-loop active power small signal model of the system is established, and the influence of transient electromagnetic power compensation on the power stability of VSG is analyzed based on root locus method. By removing the items which have little influence on the stability of the system in the small signal model, the order is reduced to obtain the equivalent second-order model of the system. According to the second-order model, the quantitative design criteria of the system parameters are given. The proposed transient electromagnetic power compensation strategy not only increases the transient equivalent damping of the system, but also does not affect the primary frequency modulation characteristics and will not cause large overshoot of the output active power. The experimental results are consistent with the theoretical analysis, which testify the effectiveness and correctness of the system control strategy and the model reduction method.

## Introduction

In recent years, a large number of renewable energy sources, such as solar energy and wind energy, they are connected to grid through power electronic converters, which greatly reduces the inertia level of the power grid^1,2^. Virtual synchronous generator (VSG) is widely concerned in order to compensate the inertia loss of the power system. Because VSG control system has added rotor motion loop, virtual governor loop, reactive power control loop and other links, it can imitate the output characteristics of synchronous generator, and has the ability of inertia support, frequency regulation and voltage control^3–5^. However, the introduction of inertia inevitably brings the rotor oscillation characteristics of synchronous generator. When faced with disturbance, the output active power of VSG has serious oscillation and overshoot problems^6,7^.

In order to suppress VSG transient active power oscillation, a lot of research has been done all over the world, which can be roughly divided into two categories. One is inertia matching method. In^8^, the expected inertia in process of power oscillation according to the power angle curve of synchronous generator is determined, and the Bang-Bang control strategy with alternating inertia to improve the transient characteristics of VSG is proposed. However, the inertia changes nonlinearly under this strategy, which affects the system performance. In^9^, an improved Bang-Bang control is adopt, which reduces active power overshoot and oscillation, weakens damping effect, and alleviates the adjustment contradiction between steady-state and dynamic characteristics, but still does not realize decoupling control between them. In^10^ and^4^, the adaptive control strategy of inertia is researched, the inertia changes dynamically and continuously with the rate of change of rotor angular velocity and its deviation. In^11^, the influence of damping is considered, and a control strategy with adaptive change of inertia and damping is proposed. In^12^, a flexible virtual inertia control strategy based on adaptive energy storage scheduling is proposed, which is beneficial to realize coordinated control among multiple micro-grids, but does not consider the influence of the energy storage system characteristics on virtual inertia. Adaptive control has better robustness than Bang-Bang control strategy, but the selection of correlation coefficient is more complex, and improper selection of frequency offset threshold will lead to virtual inertia value jitter in dynamic process, which will affect the stability of the system.

Different from the adaptive inertia method, another way to improve the transient characteristics of VSG is to introduce damping power. In^13^, The damping power control method based on phase locked loop (PLL) is adopted, which can simulate the damping mechanism of synchronous generator well. However, the frequency fluctuation of power grid in weak power grid will aggravate the output power oscillation of VSG and reduce the stability of the system. In^14^, in order to avoid the problems caused by the PLL and simplify the system structure, the actual grid frequency is replaced by the rated frequency. The damping coefficient has the same effect as the sag coefficient in the above two methods, and the introduction of damping coefficient will lead to the steady-state deviation of VSG output power. For this reason, in^15^ and^16^, the state feedback damping method is proposed, which can configure poles arbitrarily through state feedback, so as to optimize the damping and oscillation frequency of the system, but there are too many parameters to be adjusted, which makes it complicated to realize. In^17^, the mechanism of VSG synchronization loss is analyzed in detail, and a transient damping is proposed, which can effectively avoid the conflict between transient stability and frequency stability. In^18^, a transient damping lifting method based on high pass filter (HPF) is proposed, which achieved good frequency fluctuation suppression effect, but the control effect of this method was greatly affected by the filter time constant. In^19^, the method of use the first-order lag link to construct transient damping is proposed, which reduced the order of active power system by one order. In^20^, this method is applied to suppress active power oscillation of VSG parallel micro-grid, but there is a large overshoot in VSG output active power under the transient damping strategy based on the first-order lag link^21^. In^22^, a strategy of grid-connected control of VSG with virtual impedance is proposed, by introducing a virtual impedance link in the VSG electrical part, the VSG output impedance is inductive, which is helpful for the decoupling of the reactive power and the active power of the system. However, the design method of the virtual impedance value is not given. In^23^, a novel two-stage frequency-regulating control is designed for modern power systems considering high renewable energy sources (RESs) penetration and electric vehicles (EVs), and a new optimization algorithm (artificial hummingbird algorithm) is proposed to fine-tune the parameters of frequency controllers as well as VSG parameters in a low-inertia smart power system. Compared with the conventional control, the control effect is better, but the control flow is complicated.

The VSG power stabilization control methods proposed in above literatures can generally achieve good control results in some aspects such as suppressing active power oscillation or reducing active power overshoot, but the in-depth analysis of multi-objective cooperative VSG power stabilization control is still insufficient. In view of the above problems, an improved VSG control strategy with transient electromagnetic power compensation and a system order reduction method are proposed in this paper. The system can be equivalent to a second-order model for parameter design, which can not only increase transient equivalent damping to suppress power oscillation without affecting primary frequency modulation characteristics, but also alleviate output active power overshoot. Firstly, the contradiction between the dynamic and steady-state characteristics of VSG output power is analyzed. Then the transient electromagnetic power compensation is introduced to improve VSG control, and the closed loop active small signal model of the system is established. The root locus method is used to analyze the influence of transient electromagnetic power compensation on VSG power stability. Finally, according to the conclusion of root locus analysis, the items which have little influence on the stability of the system in the small signal model are removed, and the equivalent second-order model of the system is obtained by reducing the order. According to the second-order model, the quantitative design criteria of system parameters are given.

## VSG control and active power oscillation problem

### Typical topology and control of VSG

**Figure 1:**
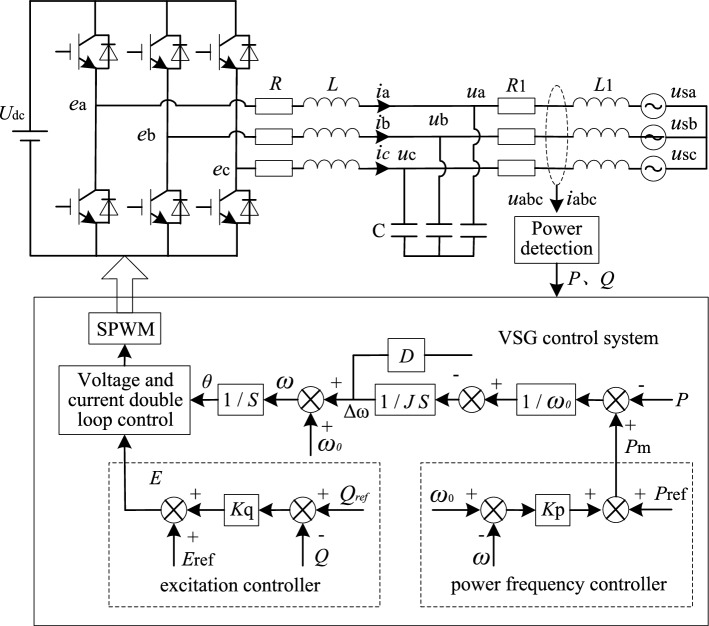
Typical circuit and control structure of VSG.

The main circuit and control system structure of VSG are shown in Fig. 1. *U*_dc_ is the DC side voltage, *L* is the filter inductor, *C* is the filter capacitor, *u*_abc_ is the filtered three phase voltage, and *i*_abc_ is the filtered three phase current. Grid-connected line impedance includes resistance *R*_1_ and inductance *L*_1_.

VSG simulates the inertia and damping characteristics of synchronous generator by introducing the rotor motion equation of traditional synchronous generator into the control system. The expression of rotor motion equation is indicated as (1).

1 $$ \frac{{P_{{m}} - P_{{e}} }}{{\omega_{0} }} - D(\omega - \omega_{0} ) = J\frac{{{\text{d}}\omega }}{{{\text{d}}t}} $$

where *P*_m_ is the input power, corresponding to the mechanical power of synchronous generator. *P*_e_ is the output active power, corresponding to the electromagnetic power of synchronous generator. *ω* is the actual angular frequency, *ω*_0_ is the rated angular frequency, *J* is the moment of inertia, and *D* is the damping coefficient.

At the same time, VSG simulates the droop characteristics of excitation regulator and governor of synchronous generator, which makes the inverter have the functions of primary frequency regulation and primary voltage regulation in grid-connected mode. Active power–frequency and reactive power–voltage sag equations are represented as (2) and (3).

2 $$ P_{{m}} = P_{{{ref}}} + K_{{P}} (\omega_{0} - \omega ) $$

3 $$ E = E_{{0}} + K_{{q}} (Q_{{{\text{ref}}}} - Q_{{\text{e}}} ) $$

where *P*_ref_ is the active power scheduling command value, *Q*_ref_ is the reactive power scheduling command value, *Q*_e_ is the actual reactive power output by VSG, *E* is the output voltage amplitude, *E*_0_ is the reference voltage amplitude, *K*_p_ is active power–frequency sag coefficient, and *K*_q_ is reactive power-voltage sag coefficient.

When the impedance of the line is inductive, active power and reactive power can be fully decoupled. For the convenience of analysis, the following contents of this paper are carried out under the condition of inductive line. In addition, because this paper focuses on active power oscillation and considers active and reactive power decoupling, the reactive power is no longer discussed in the follow up content.

### Active oscillation of VSG

**Figure 2:**
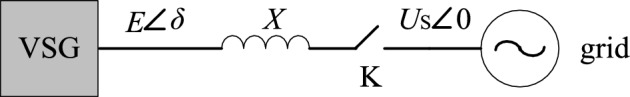
Simplified grid-connected structure of VSG.

The simplified structure of VSG grid-connected circuit is shown in Fig. 2. *E* is the amplitude of the output voltage of the inverter, *δ* is the phase angle of the output voltage, *U*_S_ is the amplitude of power grid voltage, and *X* is the equivalent output reactance of VSG.

As shown in Fig. 2, the output electromagnetic power of the VSG can be represented as follows.

4 $$ P_{{\text{e}}} = \frac{{EU_{{\text{S}}} }}{X}\sin \delta $$

Generally, *δ* is very small, (4) can be described as follows.

5 $$ P_{{\text{e}}} = \frac{{EU_{{\text{S}}} }}{X}\delta = K\delta = K\frac{{\omega - \omega_{{\text{S}}} }}{s} $$

where *K* is the synchronous voltage coefficient and *ω*_s_ is the angular frequency of grid voltage.

**Figure 3:**
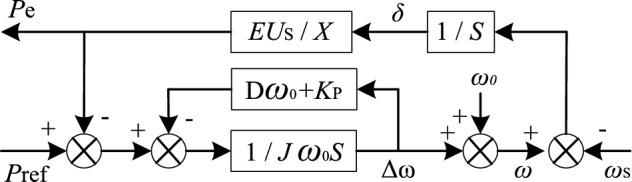
Active power closed loop control block diagram of VSG.

Because the response speed of voltage and current double inner loop control is much faster than that of power outer loop, the influence of double inner loop dynamic process on outer loop can be ignored. According to (1), (2) and (4), the grid-connected VSG active power closed loop control block diagram is shown as Fig. 3.

As shown in Fig. 3, the damping coefficient and primary frequency modulation coefficient of VSG are parameters of one effect, and there is a coupling problem between them. In addition, *P*_e_ is affected by grid frequency and active power command value, and the closed loop transfer function of *P*_e_ is represented as follows.

6 $$ P_{{\text{e}}} = \frac{{KP_{{{\text{ref}}}} }}{{J\omega_{0} s^{2} + (D\omega_{0} + K_{{\text{p}}} )s + K}} + \frac{{(J\omega_{0} s + D\omega_{0} + K_{{\text{p}}} )K(\omega_{0} - \omega_{{\text{S}}} )}}{{J\omega_{0} s^{2} + (D\omega_{0} + K_{{\text{p}}} )s + K}} $$

Based on (6), the VSG steady state output active power *P*_e0_ is described as follows.

7 $$ P_{{{\text{e0}}}} = P_{{{\text{ref}}}} + (D\omega_{0} + K_{{\text{p}}} )(\omega_{0} - \omega_{{\text{S}}} ) $$

Based on (7), the steady-state deviation of VSG output active power is (*Dω*_0_ + *K*_p_) (*ω*_0_ − *ω*_S_). For large grid with good power quality, generally *ω*_S_ = *ω*_0_, the steady-state deviation of VSG output power is 0. For large grid ends or micro-grids with low power quality, *ω*_S_ may not be equal to *ω*_0_, and there is a power deviation (*Dω*_0_ + *K*_p_) (*ω*_0_ − *ω*_S_) in steady-state. In addition, the grid frequency at the end of large grid or micro-grid generally fluctuates slowly, and can be regarded as a fixed value relative to the power loop regulation speed. Therefore, the active power transient process of grid-connected VSG is mainly affected by the step disturbance of active power command value, and the grid frequency mainly affects the steady-state accuracy of VSG.

According to (6), the output active power small signal model of VSG when adjusting active power command value in response to grid disturbance is described as follows.

8 $$ \frac{{\Delta P_{{\text{e}}} }}{{\Delta P_{{{\text{ref}}}} }} = \frac{K}{{J\omega_{0} s^{2} + (D\omega_{0} + K_{{\text{p}}} )s + K}} $$

**Figure 4:**
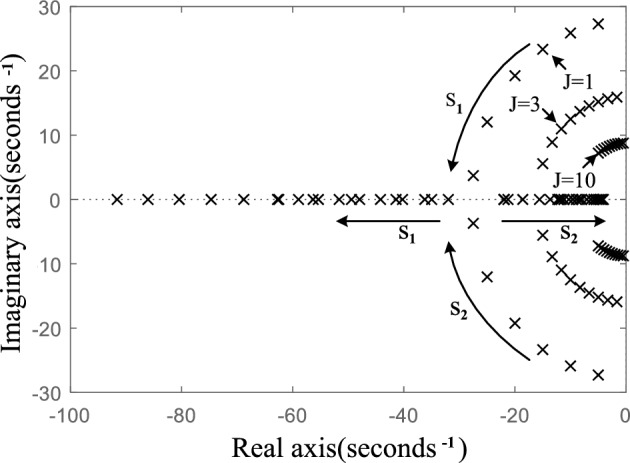
Pole distribution of active power closed loop small signal model of VSG with different J when D increases.

This is a typical second-order system. When the damping ratio of the system is less than 1, the oscillation problem will occur. Figure 4 shows the pole distribution of the system when the inertia *J* is 1, 3 and 5, respectively, and the damping *D* varies from 0 to 50. The parameters used in the drawing are shown in Table 1. *E* is the amplitude of the output voltage of the inverter, *U*_S_ is the amplitude of power grid voltage, *K*_p_ is active power–frequency sag coefficient, Δ*P*_ref_ is the increment of grid-connected active power instruction value, *J* is the moment of inertia, *D* is the damping coefficient, *X* is the equivalent output reactance of VSG, *ω*_0_ is the rated angular frequency. The parameters used in other theoretical analysis parts of this paper are shown in Table 1 unless otherwise specified.

**Table 1:** Main parameters of theoretical analysis.

Parameter	Value	Parameter	Value
*E*/V	311	*J*/(kg/m^2^)	1
*U*_S_/V	311	*D*/(N·s/rad)	0
*K*_p_/(rad/W)	2000	*X*/Ω	0.6
Δ*P*_ref_/W	4000	*ω*_0_/(rad/s)	314

According to Fig. 4, the larger the introduced inertia *J*, the closer the pole is to the imaginary axis, and the more unstable the output active power response of VSG is when the active power command value suddenly changes in response to grid disturbance. With the increase of *D*, the conjugate poles s_1_ and s_2_ of the system gradually move away from the imaginary axis and approach the real axis, and finally become two different negative real poles, the system changes from underdamping to overdamping, and the stability of the system becomes better. It shows that damping *D* can improve the stability of the system and restrain the oscillation.

To sum up, for the typical grid-connected VSG system shown in Fig. 1, the existence of inertia *J* makes its output active response behave as a second-order oscillation system, and the larger *J*, the more intense the oscillation. Damping *D* can effectively suppress this oscillation, but *D* is coupled with the primary frequency modulation parameter. According to (7), *D* will increase the output active steady-state deviation. The above analysis results show that there is a certain contradiction between the dynamic and static performance of VSG, it is difficult to suppress active power oscillation by designing VSG control parameters directly.

## Improved VSG control with transient electromagnetic power compensation

In order to suppress the output active power oscillation in grid-connected VSG without affecting the steady-state output power, an improved VSG control strategy based on transient electromagnetic power compensation is proposed in this section.

### Improved VSG control strategy

**Figure 5:**
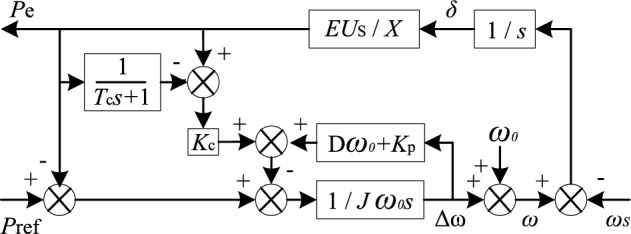
Active power control closed-loop block diagram of improved VSG.

The proposed improved VSG control strategy introducing transient electromagnetic power compensation is shown in Fig. 5. Based on conventional VSG active power control, this strategy adds an electromagnetic power passing through the lag link, and makes a difference between it and the initial electromagnetic power to get the transient power difference. Multiply the obtained transient power difference by the compensation coefficient Kc and add it to the power control, so as to form transient electromagnetic power compensation in the process of power fluctuation and achieve the effect of suppressing oscillation.

The proposed improved VSG control strategy adds a transient power to the original VSG active power control. The rotor motion equation of improved VSG is shown in (9).

9 $$ P_{{\text{m}}} - P_{{\text{e}}} - D\omega_{0} (\omega - \omega_{{0}} ) - P_{{\text{c}}} = J\omega_{0} \frac{{{\text{d}}\omega }}{{{\text{d}}t}} $$

where *P*_c_ is the introduced transient compensation power, its value is composed of the first-order hysteresis electromagnetic power and the original electromagnetic power. It is described by (10).

10 $$ P_{{\text{c}}} = K_{{\text{c}}} \left( {1 - \frac{1}{{T_{{\text{c}}} s + 1}}} \right)P_{{\text{e}}} $$

where *T*_c_ is the time constant of the first-order lag link in compensating electromagnetic power. *K*_c_ is the corresponding compensation coefficient.

Because this power difference or angular frequency difference only exists in transient process, it will not affect the steady-state performance. As can be seen from Fig. 5, the output active power small signal model of the improved VSG is expressed as (11).

11 $$ \frac{{\Delta P_{{{\text{ec}}}} }}{{\Delta P_{{{\text{ref}}}} }} = \frac{{K(T_{{\text{c}}} s + 1)}}{{T_{{\text{c}}} J\omega_{0} s^{3} + ms^{2} + ns + K}} $$

where

$$ \left\{ \begin{gathered} m = J\omega_{0} + T_{c} (D\omega_{0} + K_{{\text{p}}} ) \hfill \\ n = D\omega_{0} + K_{{\text{p}}} + KT_{{\text{c}}} (K_{{\text{c}}} + 1) \hfill \\ \end{gathered} \right. $$

Comparing (11) with (8), it is found that the improved VSG active power system adds a pole and a zero and changes the original two poles due to the introduction of transient electromagnetic compensation power, which will change the response characteristics of the system and suppress the power oscillation through reasonable adjustment.

### Analysis of the influence of improved strategy on VSG power stability and overshoot

(11) is arranged as follows.

12 $$ \frac{{\Delta P_{{{\text{ec}}}} }}{{\Delta P_{{{\text{ref}}}} }} = \frac{{K(T_{{\text{c}}} s + 1)}}{{\alpha (T_{{\text{c}}} s + 1) + KT_{{\text{c}}} K_{{\text{c}}} s}} $$

where *α* = *Jω*_0_*s*^2^ + (*Dω*_0_ + *K*_p_)*s* + *K*.

Comparing (12) with (8), it shows that if *KT*_c_*K*_c_ is very small, (12) and (8) can be approximately equal, it indicates that the effect of the improved VSG compensation using transient electromagnetic power compensation is not obvious. The *KT*_c_*K*_c_ value should not be too small for the proposed compensation strategy to have a significant impact on the power stability of VSG. According to (5), it can be seen that the value of the synchronization voltage coefficient *K* is generally large. Therefore, the *T*_c_*K*_c_ value of the improved VSG need not be very large to affect the oscillation process. According to the lag characteristic, the lag time constant should be smaller in order to ensure the timeliness of transient compensation link response and rapid failure in steady-state.

**Figure 6:**
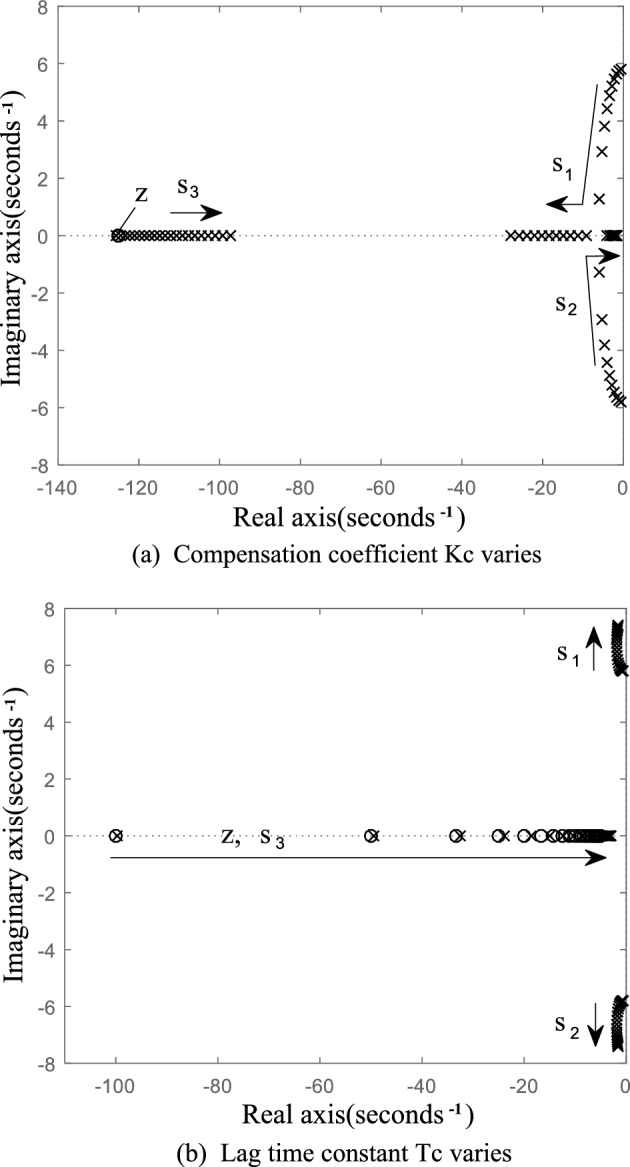
Pole-zero distribution of active power closed loop small signal model of the improved VSG when the parameters of the compensation link vary.

Figure 6a shows the zero-pole distribution of the improved VSG system when the lag time constant *T*_c_ = 0.006 and the compensation coefficient *K*_c_ increases from 0 to 30. Figure 6b shows the zero-pole distribution of the improved VSG active power response when the lag time constant *T*_c_ changes from 0 to 2, where *K*_c_ = 1.

As shown in Fig. 6a, the improved VSG system has one more zero and one more pole than the conventional VSG system. When *K*_c_ varies from 0 to 30, the increased negative real pole s_3_ is close to the imaginary axis, but always far away from it. With the increase of *K*_c_, the dominant pole pair s_1_ and s_2_ quickly move away from the imaginary axis and approach the real axis from the original position. With the further increase of *K*_c_, they become two real poles. Real pole s_2_ near the imaginary axis becomes the dominant pole. It indicates that the introduced *K*_c_ improves the equivalent damping of the system and enhances the stability of the system. With the increase of *K*_c_, the system will change from under-damping to over-damping, but the value of *K*_c_ should not be very large. If it is very large, the dominant pole will be infinitely close to the imaginary axis, which will slow down the response and lengthen the response time. The improved VSG pole change shown in Fig. 6a testify the effectiveness of the transient electromagnetic power compensation strategy proposed in this paper.

As shown in Fig. 6b, when the lag time constant *T*_c_ increases from 0 to 2, the dominant poles s_1_ and s_2_ of the improved VSG system move away from the original position, but the distance from the imaginary axis is basically unchanged, which represents that the lag time constant has little influence on the power stability of the improved VSG system. At the same time, it should be noted that the improved VSG has a pole which is very close to the zero z when the lag time constant *T*_c_ varies, and they form a pair of dipoles, which have little influence on the stability of the system. However, it can be found that when the delay time constant *T*_c_ is large, the pair of zeros and poles is very close to the imaginary axis, which may seriously affect the stability of the system, it further shows that the delay time constant *T*_c_ should be a small value.

## Model reduction and parameter design

According to the conclusion of root locus qualitative analysis in the previous section, this section reduces the order of small signal model, and obtains the equivalent second-order model of VSG with transient electromagnetic power compensation, so that the system parameters can be quantitatively designed according to the equivalent second-order model.

### Small signal model order reduction

According to (11), the transient electromagnetic power compensation is introduced, which has an effect on the stability of the system and increases the order of the system. As can be seen from Fig. 6, *T*_c_ has little influence on the location of the dominant pole pair, and the location change of the dominant pole of the system is mainly affected by *K*_c_. It indicates that the stability of the system is mainly affected by the change of compensation coefficient *K*_c_, the lag time constant *T*_c_ has little influence. Based on this analysis, this paper presents a simplified method to reduce (11) to a second-order system. All the terms containing *T*_c_ but not *K*_c_ in the numerator and denominator of (11) are removed. The equivalent second-order system is shown in (13).

13 $$ \frac{{\Delta P_{{{\text{ec}}}}{\prime} }}{{\Delta P_{{{\text{ref}}}}{\prime} }} = \frac{K}{{J\omega_{0} s^{2} + (D\omega_{0} + K_{{\text{p}}} + KT_{{\text{c}}} K_{{\text{c}}} )s + K}} $$

**Figure 7:**
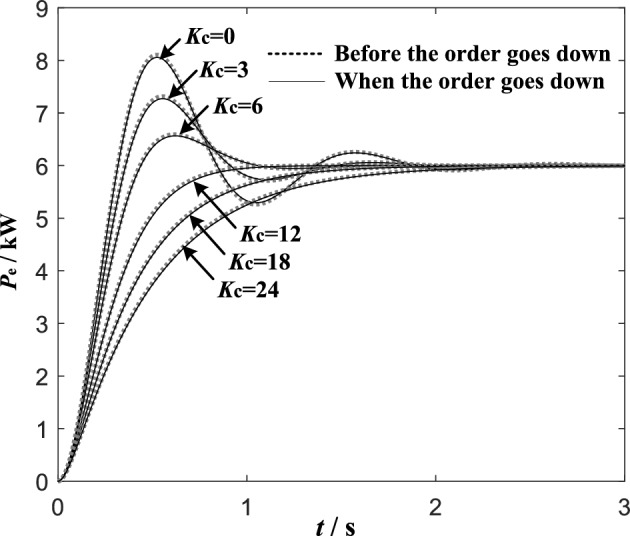
Comparison of step response of transient electromagnetic power compensation VSG active closed loop small signal model before and after order reduction.

To demonstrate the effectiveness of the simplified method proposed in this paper, the comparison of the step response of (11) and (13) with different *K*_c_ is shown in Fig. 7.

**Figure 8:**
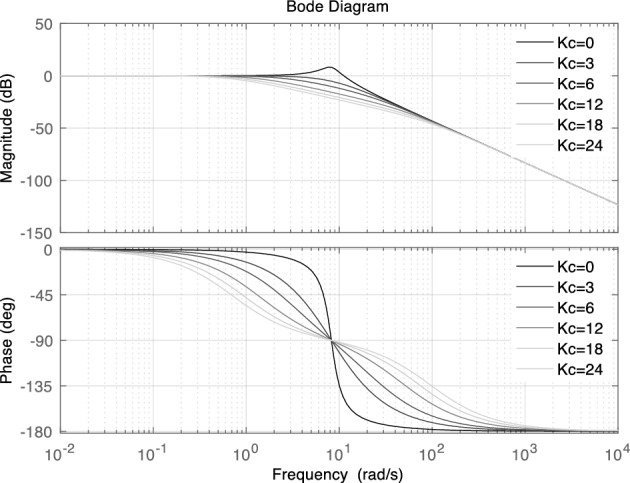
Bode diagram of VSG active power closed loop control system.

As can be seen from Fig. 7, the step responses of the second-order model and the third-order model under different *K*_c_ are very consistent, there only slight deviation. It indicates that the simplified method is effective, the influence of compensation parameters on the power response before and after order reduction is very consistent. So, it is reasonable to design parameters according to the simplified second-order model. Figure 8 is the bode plots of the closed loop systems.

### Parameter design

According to the simplified small signal model, the parameters can be further tuned from the angle of damping ratio and phase margin of the second-order system. Based on (13), the natural oscillation frequency *ω*_n_ and damping ratio *ζ* of the system are shown in (14) and (15).

14 $$ \omega_{n} = \sqrt {\frac{K}{{J\omega_{0} }}} $$

15 $$ \zeta = \frac{{D\omega_{0} + K_{{\text{p}}} + KT_{{\text{c}}} K_{{\text{c}}} }}{{2\sqrt {KJ\omega_{0} } }} $$

According to the control theory, the phase margin *γ* and cut-off frequency *ω*_c_ of (13) can be represented as follows.

16 $$ \gamma = \arctan \frac{2\xi }{{\sqrt {\sqrt {1 + 4\xi^{2} } - 2\xi^{2} } }} $$

17 $$ \omega_{{\text{c}}} = \omega_{{\text{n}}} \sqrt {\sqrt {1 + 4\xi^{2} } - 2\xi^{2} } $$

**Figure 9:**
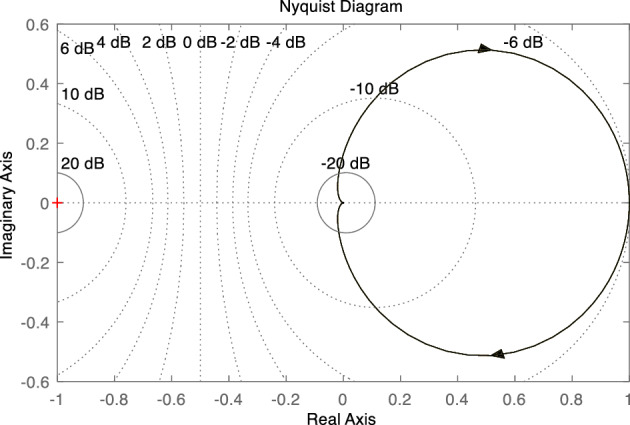
Bode diagram of VSG active power closed loop control system.

In order to make the control system stable, the damping ratio should not be too small. Generally, 0.8 < *ζ* < 1, and the phase margin should not be less than 45°. As shown in Fig. 9, the Nyquist diagram of the system does not intersect the negative real axis, indicating that the amplitude margin of the system is infinite.

## Experiment verification

**Figure 10:**
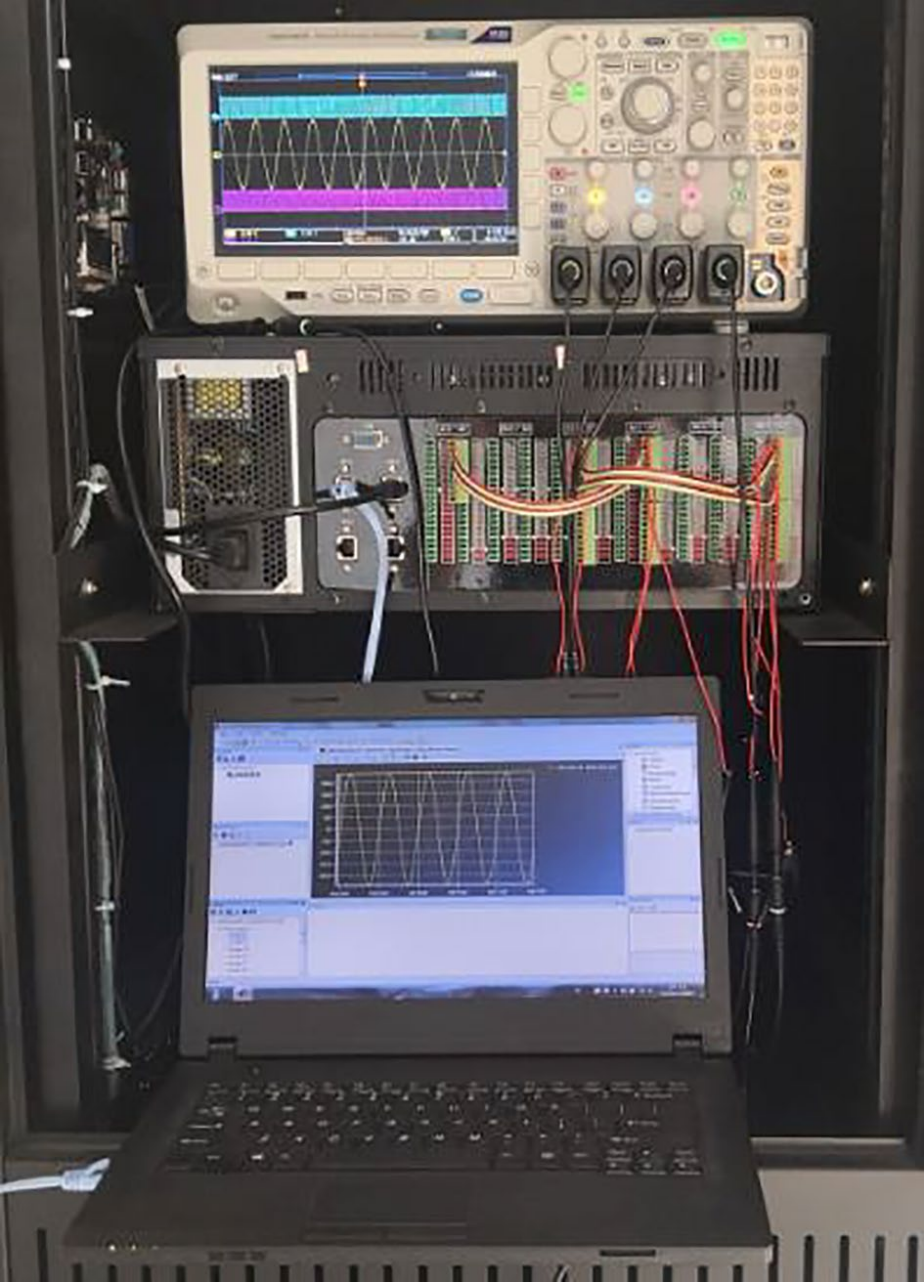
Hardware-in-loop experiment system.

In order to verify the correctness and effectiveness of the proposed control strategy, a VSG grid-connected controller level hardware-in-loop test system is built as shown in Fig. 10.

It consists of upper computer, real time simulator (HBUREP-100), DSP controller (TMS320F28335), and oscilloscope. The conventional VSG control strategy and the improved VSG control strategy proposed in this paper are used to build the VSG system model on the upper computer according to the topology shown in Fig. 1, and the model is compiled and generated into C code, which is loaded into the real time simulator to run. The real time simulation model in the simulator includes two parts, the main circuit and the control system. TCP/IP network communication protocol is used to transmit information between simulator and host computer. DSP and simulator are directly connected through A/D and D/A conversion interfaces to realize high speed signal transmission. PWM pulses generated by DSP operation are connected with pulse input port of simulator through photoelectric isolation module. The main parameters of the system are shown in Table 2. *U*_d_ is the rated voltage of the DC bus, *E*_0_ is the reference voltage amplitude, *P*_ref_ is the active power scheduling command value, *K*_p_ is active power–frequency sag coefficient, *ω*_0_ is the rated angular frequency, Δ*P*_ref_ is the increment of grid-connected active power instruction value, *J* is the moment of inertia, *D* is the damping coefficient, *T*_c_ is the time constant of the first-order lag link in compensating electromagnetic power, *R* is the internal resistance of the filter inductance, *L* is the filter inductor, *C* is the filter capacitor, *g*rid-connected line impedance includes resistance *R*_1_ and inductance *L*_1_. At *t* = 3s, the active power dispatching instruction of grid-connected VSG is increased by 4kW. In order to reflect the steady-state deviation caused by the difference between VSG rated frequency and grid frequency, the actual frequency of grid is set to 49.97 Hz.

**Table 2:** Main parameters of experiment.

Parameter	Value	Parameter	Value
*U*_d_/V	650	*D*/(N·s/rad)	0
*E*_0_/V	311	*T*_c_/s	0.005
*P*_ref_/W	6000	*R*/Ω	0.1
*K*_p_/(rad/W)	2000	*L*/mH	10
*ω*_0_/(rad/s)	314	*C*/μF	30
Δ*P*_ref_/W	4000	*R*_1_/Ω	0.3
*J*/(kg/m^2^)	1	*L*_1_/mH	15

### Traditional VSG control strategy

**Figure 11:**
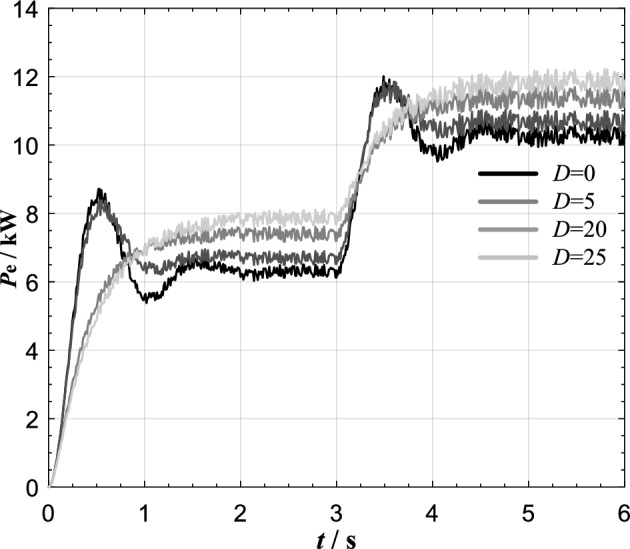
Simulation results of fixed damping strategy.

The experimental results using conventional VSG control strategy are shown in Fig. 11.

The results show that when *D* = 0, the active power command value step at *t* = 3 s causes the VSG output active power oscillation lasting about 2 s, and the active power overshoot reaches 1.8 kW. After damping *D* is added, the active oscillation and overshoot are obviously suppressed. And the greater the damping *D*, the more remarkable the suppression effect is. On the other hand, due to the difference between VSG rated frequency and grid frequency, there is a steady-state deviation (*Dω*_0_ + *K*_p_)(*ω*_0 _− *ω*_S_) in VSG output active power. When D is assigned as 0, 5, 15 and 25, the corresponding steady-state deviation is about 400, 714, 1342 and 1656W respectively. It can be seen that when the conventional VSG control strategy is adopt, the active oscillation can be effectively suppressed under the action of damping. With the increase of damping, the active overshoot is obviously reduced, but at the same time, the steady-state power deviation is also increased.

### Transient electromagnetic power compensation strategy

**Figure 12:**
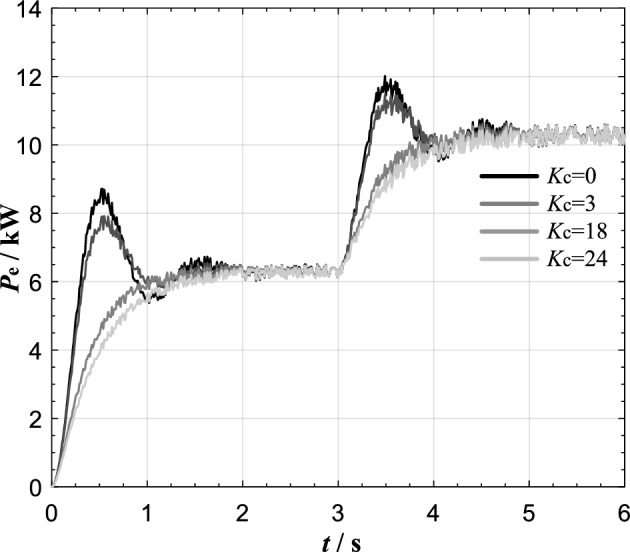
Simulation results of transient electromagnetic power compensation strategy.

The experimental results of VSG active oscillation suppression using the transient electromagnetic power compensation method proposed in this paper are shown in Fig. 12.

The results show that when the compensation coefficient Kc = 0, the active power command value step at *t* = 3 s causes the VSG output active power oscillation lasting about 2 s, and the active power overshoot reaches 1.8 kW. After compensating the electromagnetic power, the oscillation is obviously suppressed. With the increase of compensation coefficient *K*_c_, the active oscillation and overshoot of VSG output become smaller and smaller. When the system enters the over damping state, with the further increase of the compensation coefficient *K*_c_, the response time of the system will increase obviously. At the same time, because this power compensation only exists in transient process, it does not cause steady-state deviation. The simulation results are completely consistent with the theoretical analysis results in Fig. 6a. It indicates that when the proposed transient electromagnetic power compensation strategy is adopt, with the increase of compensation coefficient *K*_c_, the active power oscillation of grid-connected VSG can be better suppressed. The *K*_C_ value can be adjusted to achieve the control purpose of neither overshoot nor steady-state deviation.

## Conclusion

In view of the problem of active power oscillation of VSG under power grid disturbance, a VSG control strategy with transient electromagnetic power compensation is proposed in this paper. Through theoretical analysis and experimental verification, the following conclusions are obtained.

1. The proposed transient electromagnetic power compensation strategy can effectively suppress the VSG power oscillation. The transient electromagnetic power compensation increases the transient equivalent damping of the system, and does not affect the primary frequency modulation characteristics. At the same time, the zero pole caused by transient electromagnetic power compensation is far away from the imaginary axis, so the output active power overshoot will not be caused.
2. By removing the items that have little influence on the stability of the system in the small signal model, the order of the improved VSG active power system is reduced, the equivalent second-order model is obtained, and the quantitative design method of system parameters is given.

The research in this paper provides some theoretical and methodological supports for the large-scale friendly grid connection of new energy, especially for its active defense against grid disturbance.

All data generated or analysed during this study are included in this published article.
